# The Feasibility and Safety of Prehospital Whole Blood Administration for Patients in Hemorrhagic Shock in Isolated Regions of Colorado: Assessment of the First 6 Months

**DOI:** 10.1002/wjs.70277

**Published:** 2026-02-20

**Authors:** Matthew Branney, Sydney Mattox, John Lynch, Scott Branney, Laura Harwood, Rebecca Ryznar, Zsolt J. Balogh

**Affiliations:** ^1^ College of Osteopathic Medicine Rocky Vista University Englewood Colorado USA; ^2^ Colorado Whole Blood Coalition Highlands Ranch Colorado USA; ^3^ CommonSpirit St. Anthony Hospital Lakewood Colorado USA; ^4^ Department of Biomedical Sciences Rocky Vista University Englewood Colorado USA; ^5^ Division of Surgery, Department of Traumatology John Hunter Hospital and University of Newcastle Newcastle New South Wales Australia

## Abstract

The aim of this pilot study is to summarize evidence from the implementation of prehospital whole blood programs in rural Colorado with focus on the feasibility and safety of whole blood in rural areas with prolonged transport times to definitive care.

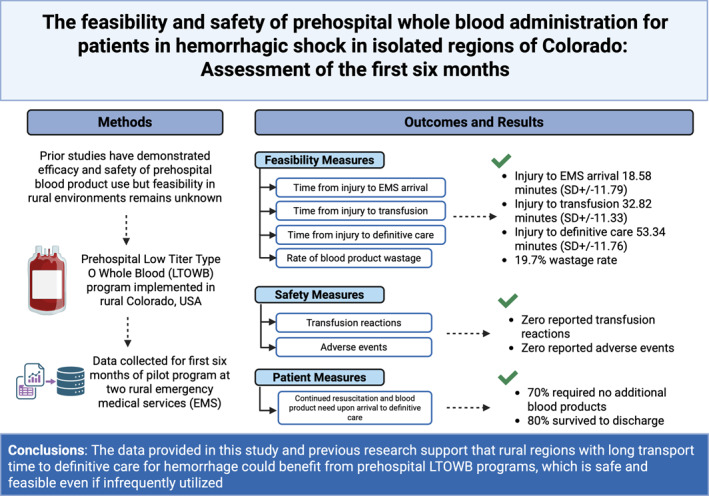

## 1 Introduction

Bleeding is the leading cause of preventable postinjury deaths for which reducing the time between onset and definitive control of hemorrhage is critical for reducing mortality [1]. The current management of traumatic hemorrhage includes the avoidance and reversal of hypoperfusion with blood products when available and pharmacologic adjuncts to deliver timely balanced initial resuscitation [2]. Low titer type O whole blood (LTOWB) has been demonstrated as a safe and effective resuscitation strategy as a single blood product in the treatment of hemorrhagic shock, mitigating the financial and logistic constrains of carrying multiple blood products to deliver hemostatic resuscitation [3]. Most prehospital whole blood in the United States is administered via air ambulance and a limited number of ground ambulances, mostly operating in urban and metropolitan areas [4]. Feasibility and safety data for blood programs primarily serving rural areas remain limited.

The aim of this pilot study is to summarize evidence from the implementation of prehospital whole blood programs in rural Colorado with focus on the feasibility and safety of whole blood in rural areas with prolonged transport times to definitive care.

## 2 Methods

This retrospective study evaluated all prehospital LTOWB administrations from December 25, 2024, to June 25, 2025, across Grand County (GCEMS) and Clear Creek County (CCEMS) after implementation of a prehospital whole blood program in rural regions of Colorado.

Program establishment and protocols for LTOWB administration are detailed in Supporting Information S1 and S2, respectively. Rocky Vista University College of Osteopathic Medicine's Institutional Review Board approved the study (IRB # 2025‐062).

Feasibility variables included time from EMS activation to arrival, transfusion and definitive care, EMS arrival to transfusion, and the rate of blood product wastage. Safety outcomes included transfusion reactions and adverse events. Pertinent hemodynamic variables were compared before and after transfusion. Lastly, we analyzed variables obtained at the time of arrival to definitive care including need for emergent hemorrhage control, continued resuscitation needs, and patient disposition.

Descriptive and summary statistics were used to identify mean and standard deviation of continuous variables, median, and interquartile range (IQR) of categorical variables. All binary variables were evaluated and reported as proportions in percentages.

## 3 Results

During the study period, CCEMS responded to 1036 EMS activations and provided LTOWB to six patients, whereas GCEMS responded to 779 EMS activations and provided LTOWB to four patients, transfusing LTOWB in 0.58% and 0.51% of activations, respectively. Participant demographics are presented in Table 1.

**TABLE 1:** Participant demographic variables including age (years), sex, race, and etiology of hemorrhagic shock.

Participant demographics		
Age (years)	*n* (10)	%
< 18	1	0.10
19–29	3	0.30
30–39	1	0.10
40–49	0	0.00
50–59	0	0.00
> 60	5	0.50
Sex	*n* (10)	%
Female	4	0.40
Male	6	0.60
Race	*n* (10)	%
White	8	0.80
Hispanic	2	0.20

Hemorrhage etiology		
	*n* (6)	%
Traumatic (total)	6	0.60
Penetrating	1	0.16
Blunt	3	0.50
Combined	2	0.33
	*n* (4)	%
Nontraumatic (total)	4	0.40
Upper gastrointestinal bleeding	3	0.75
Lower gastrointestinal bleeding	1	0.25

Mean time from EMS activation to arrival was 18.58 min (SD ± 11.79 min), EMS activation to transfusion was 32.82 min (SD ± 11.33 min), EMS activation to definitive care was 53.34 min (SD ± 11.76 min), and EMS arrival to transfusion was 15.28 min (SD ± 9.95 min). Two interagency rendezvous resulted in transport delays of 0.75 and 2.15 min. Neither transfusion reactions nor adverse events were reported.

Seventy one units of LTOWB were distributed, 59 were returned after 14 days of which 45 were transfused before expiration yielding a 19.7% wastage rate. No units were removed from service following inadequate temperature control, resulting in zero prehospital wastage. Participant hemodynamic variables, resuscitation needs, and disposition variables are presented in Table 2.

**TABLE 2:** Participant variables including LTOWB utilization by county, transport method by county and pre‐ and post‐LTOWB administration hemodynamic and physiologic measures separated by etiology of hemorrhage.

	Grand County (*n* = 4)		Clear Creek County (*n* = 6)		Total (*n* = 10)	
	*n*	%	*n*	%	*n*	%
Participant variables						
LTOWB utilization						
Patients receiving 1‐unit LTOWB	3	0.75	6	1.00	9	0.90
Patients receiving > 1‐unit LTOWB	1	0.25	0	0.00	1	0.10
Transport method						
Air ambulance only	0	0.00	0	0.00	0	0.00
Ground ambulance only	1	0.25	6	1.00	7	0.70
Ground + air ambulance	3	0.75	0	0.00	3	0.30

Patients with traumatic etiology of hemorrhage (*n* = 6)					Patients with medical etiology of hemorrhage (*n* = 4)				
	Pre‐LTOWB		Post‐LTOWB			Pre‐LTOWB		Post‐LTOWB	
	Median	IQR	Median	IQR		Median	IQR	Median	IQR
Injury Severity Score (ISS)	53.5	44.0	NA	NA					
Glasgow Coma Scale (GCS)	9.5	8.0	3.0^a^	11.0^a^	Glasgow Coma Scale (GCS)	14.0	1.15	15	0.00

	Mean	SD	Mean	SD		Mean	SD	Mean	SD
Shock index (SI)	1.72	0.97	1.12	0.38	Shock index (SI)	1.05	0.23	0.83	0.12
Heart rate (HR)	93.66	49.38	83.33	43.93	Heart rate (HR)	111.25	10.56	101.00	7.87
Systolic blood pressure (SBP; mmHg)	70.67	55.88	80.50	49.07	Systolic blood pressure (SBP; mmHg)	110.00	30.38	123.75	20.47
Respiratory rate	22.83	14.73	17.17^a^	9.43^a^	Respiratory rate	18.75	4.42	17.50	1.91
Oxygen saturation (SpO_2_)	67.33	31.58	NA	NA	Oxygen saturation (SpO_2_)	90.75	7.46	NA	NA
End‐tidal carbon dioxide (EtCO_2_; mmHg)	26.25	9.14	NA	NA	End‐tidal carbon dioxide (EtCO_2_; mmHg)	17.74	8.14	NA	NA
Temperature (C)	NA	NA	34.96	3.32	Temperature (C)	NA	NA	36.60	0.36
Venous lactate (mmol/L)	NA	NA	12.12	11.67	Venous lactate (mmol/L)	NA	NA	5.28	5.73

Need for emergent hemorrhage control operation			Required additional blood products during initial inpatient resuscitation		
	*n*	%		*n*	%
Yes			Yes	3	0.30
Exploratory laparotomy	2	0.20	No	7	0.70
Thoracotomy	1	0.10			
No	7	0.70	Patient disposition		
			Survival	8	0.80
			Death	2^b^	0.20^b^

^a^ At arrival to definitive care, three patients underwent rapid sequence induction (RSI) reducing GCS to 3.0 and fixing respiratory rate at 16.0.

^b^ One patient was palliated 10 days after admission due to nonsurvivable traumatic brain injury (TBI).

## 4 Discussion

This pilot study demonstrated that prehospital administration of LTOWB in rural regions of Colorado is very infrequent, but feasible, safe, and logistically sustainable. This experience could be considered for implementation of prehospital LTOWB in other rural locations around the world.

Despite operating in resource limited environments, providers achieved transfusion times consistent with Level‐1 trauma center benchmarks of < 15 min. Neither county has Level‐1 or 2 trauma centers and both experience adverse weather conditions limiting air ambulance transport necessitating prolonged ground ambulance transport.

In addition to our promising feasibility and safety pilot, prior research suggests prehospital LTOWB reduces overall blood product usage, alleviating strain on the supply chain [5, 6]. Three patients required additional blood products during their initial inpatient resuscitation (Table 2). This association requires further investigation to assess causation and identify underlying mechanisms.

Timely reversal of traumatic shock‐associated hypoperfusion does not always lead to functional survival but the secondary benefit from the prevention of severe organ dysfunction is considerable for organ donation. Additionally, this study highlights the importance of interagency operability by demonstrating negligible increases in transport times during two transfusions conducted via rendezvous of a transporting ambulance without whole blood en route to definitive care.

Our findings of usage rates align with prior European and urban United States prehospital LTOWB studies suggesting scalability of such programs to rural systems [7, 8]. Limitations include small sample size and retrospective design which precludes evaluation of mortality benefit and the appropriateness of the indication of prehospital transfusion.

The data provided in this study and previous research support that rural regions with long transport time to definitive care for hemorrhage could benefit from prehospital LTOWB programs, which is safe and feasible even if infrequently utilized.

## Author Contributions

**Matthew Branney:** conceptualization, data curation, formal analysis, methodology, project administration, visualization, writing – original draft, writing – review and editing. **Sydney Mattox:** writing – original draft, writing – review and editing, visualization. **John Lynch:** writing – original draft, writing – review and editing. **Scott Branney:** conceptualization, data curation, investigation, project administration, supervision, writing – review and editing. **Laura Harwood:** data curation, writing – review and editing. **Rebecca Ryznar:** conceptualization, methodology, project administration, supervision, visualization, writing – original draft, writing – review and editing. **Zsolt J. Balogh:** conceptualization, methodology, project administration, supervision, visualization, writing – original draft, writing – review and editing.

## Funding

The authors have nothing to report.

## Conflicts of Interest

The authors declare no conflicts of interest.

## Supporting information


Supporting Information S1

Supporting Information S2

## Data Availability

The data that support the findings of this study are available from the corresponding author upon request. The data are not publicly available due to privacy or ethical restrictions.
