# Supplementary figures and images for: The Feasibility and Safety of Prehospital Whole Blood Administration for Patients in Hemorrhagic Shock in Isolated Regions of Colorado: Assessment of the First 6 Months
Source: World J Surg. 2026 Feb 20;50(4):1097–100. doi: 10.1002/wjs.70277 (PMC13070434; doi:10.1002/wjs.70277)

# Prehospital LTOWB Program Establishment and Maintenance

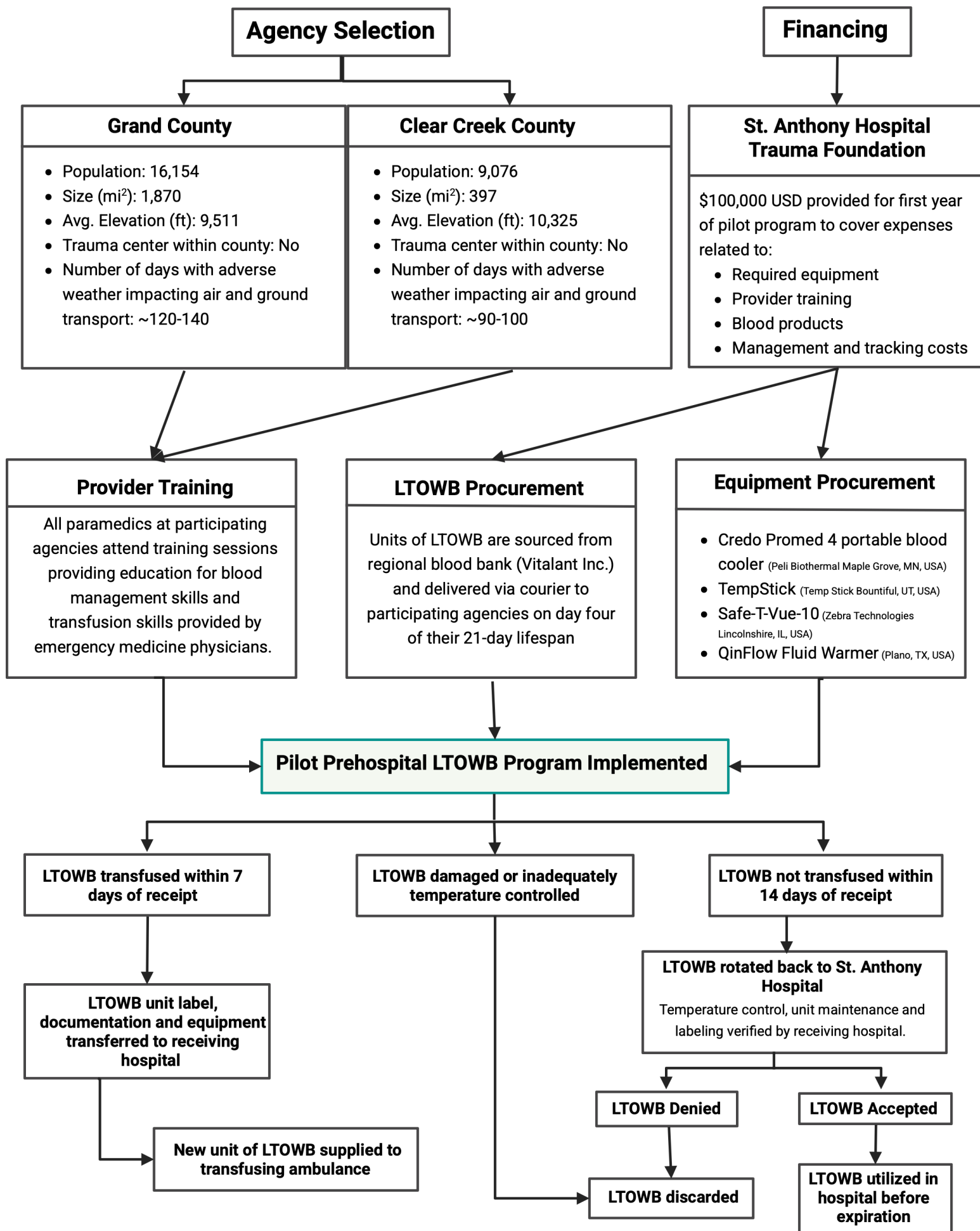

Supplement: Supplementary file 1 — Supporting Information S1 [file WJS-50-1097-s002.pdf]
