# Supplementary material for: The Feasibility and Safety of Prehospital Whole Blood Administration for Patients in Hemorrhagic Shock in Isolated Regions of Colorado: Assessment of the First 6 Months
Source: World J Surg. 2026 Feb 20;50(4):1097–100. doi: 10.1002/wjs.70277 (PMC13070434; doi:10.1002/wjs.70277)
Supplement: Supplementary file 2 — Supporting Information S2 [file WJS-50-1097-s001.pdf]

HEMORRHAGIC SHOCK – LOW TITER O WHOLE BLOOD

COLORADO WHOLE BLOOD COALITION

HEMORRHAGIC SHOCK in medical or trauma adult and pediatric patients

Hypotensive for age and/or indications of poor perfusion or hemodynamic instability

Consider other etiologies of shock state and treat reversble causes of shock (e.g., control massive external hemorrhage)

Do they meet citeria for Whole Blood?

Yes

Transfuse Blood Product:
Low Titer O Whole Blood (LTOWB)
(500mL Adults, 10mL/kg for Peds) IV/IO

Tranexamic Acid- per local protocol

Calcium- per local protocol

No

Refer to General Trauma Care protocol

Examples of Hemorrhagic Shock

- GI Bleed
- Postpartum Hemorrhage
- Vascular – uncontrolled bleeding shunt, fistula, or varicose vein
- Urological – Especially w/ recent surgery/procedure

Consider Non-Hypovolemic Causes of Shock

- Dysrhythmia, myocardial ischemia/infarct Sepsis
- Anaphylaxis
- Cyanide or Carbon Monoxide poisoning
- Pulmonary Embolism

Other causes of traumatic shock may include:

- Tension Pneumothorax
- Pericardial Tamponade
- Neurogenic

Blood Product Transfusion Criteria:

- Blood product is available
- Provider impression: Shock is believed to be due to hemorrhage (traumatic or other occult hemorrhage)
- Patient of any age
- Patient has no stated objections to blood products
- Patient has **any** of the following indicators of shock:
  - Systolic blood pressure (SBP) < 90 mmHg **OR**
  - Adult Shock Index (HR/SBP) > 0.9 **OR**
  - Modified Shock Index (HR/MAP) > 1.3 **OR**
  - Pediatric Age-Adjusted Shock Index
    - Ages 4-6: >1.2
    - Ages 7-12: >1.0
    - Ages 13-16: >0.9
  - ETCO2 < 25 **OR**
  - Witnessed traumatic arrest < 10 min PTA EMS **OR**
  - Age ≥ 65 y/o with SBP ≤ 100 mmHg **AND** HR ≥ 100 bpm

Pediatric TBI (MAP Goal)

Age	MAP
0-2 years	50-70
2-5 years	60-80
6-8 years	65-85
9+ years	70-95

Pediatric Shock Indicators

Compensated

- Normal systolic blood pressure
- Tachycardia
- Prolonged (>2 seconds) capillary refill
- Tachypnea
- Cool and pale distal extremities
- Weak peripheral pulse

Decompensated

- Weak central pulses
- Poor color
- Hypotension for age

Blood transfusion should not delay transport and should occur while enroute to the hospital. Rapid treatment and transport to a trauma facility remains priority in all cases of traumatic shock.
